# Ankyloglossia, Syndactyly and Polydactyly in the Pediatric Patient

**DOI:** 10.7759/cureus.35443

**Published:** 2023-02-25

**Authors:** Joana Morgado Dias, Ana Baptista, Nuno Alegrete, Cristina Areias, Henrique Soares

**Affiliations:** 1 Pediatric Dentistry, Oporto University, Porto, PRT; 2 Pediatric Dentistry, Cooperativa de Ensino Superior Politécnico e Universitário, Porto, PRT; 3 Pediatric Orthopedics, Hospital CUF, Porto, PRT; 4 Pediatrics, Oporto University, Porto, PRT

**Keywords:** lingual frenotomy, polydactyly, syndactyly, tongue-tie, ankyloglossia

## Abstract

Ankyloglossia is a congenital alteration in the development of the tongue characterized by the presence of a short or thick lingual frenulum, which leads to a limitation in its movements. There is an associative inconsistency between ankyloglossia and complications with breastfeeding, speech, swallowing, breathing, and the development of orofacial structures, and it is urgent to make more scientific research in this area. In the presence of polydactyly and syndactyly may be also present ankyloglossia. The purpose of this paper is to present two ankyloglossia cases with finger alterations, without a syndromic disease, and lead the medical team to research this topic and make an improved treatment plan for these cases.

## Introduction

In recent years, interest in the topics of lingual frenulum and ankyloglossia in the scientific community has increased, due to its impact on the motor function and development of the oral cavity. The American Academy of Pediatric Dentistry (AAPD) defines ankyloglossia as a congenital alteration in the development of the tongue characterized by the presence of a short or thick lingual frenulum, which leads to a limitation in its movements [1,2]. Mills et al. [3] classify the lingual frenulum as a dynamic structure formed by a fold in the fascia of the oral floor, with significant morphological variability in the fixation of this same fascia in the midline. These are distinguished by how the mucosa, fascia, and/or fibers of the genioglossus muscle are present in the lingual frenulum when lingual movements occur [4]. The prevalence of ankyloglossia in newborns varies between 1% and 32.5%, depending on the population studied, its definition and classification criteria [1,5,6].

Currently, there is an associative inconsistency between ankyloglossia and complications with breastfeeding, speech, swallowing, breathing, or the development of orofacial structures, with many published articles indicating the need for more studies of the high scientific impact of this topic [1,2,4].

As an oral surgery that involves incision and/or excision, hemostasis, and correct healing of the surgical wound, the AAPD recommends a soft diet, regular oral hygiene, and, if necessary, analgesic usage. Postoperative exercises are recommended to prevent reattachment of the wound. The use of electrosurgical or laser technology has demonstrated a shorter operative time, better bleeding management, reduced intra- and postoperative pain and discomfort, no need for sutures, and greater acceptance [1].

Despite being a relatively common disorder in newborns (0.19% in live births) [7], polydactyly can be associated with several syndromes, and it may be useful to plan a more detailed investigation of the child who presents this disorder [8]. Characterized by the presence of an extra digit, polydactyly is a congenital upper limb anomaly and there are two types of polydactyly: type A and type B. In the type A, a fully developed extra digit articulates with either the fifth metacarpal or a duplicate metacarpal. The most common being type B, a rudimentary non-functional digit that is attached by a soft tissue bridge. Approximately three-quarters of cases of type B polydactyly are bilateral, and 85% have a family history. Regarding treatment, a recent review and meta-analysis suggests that suture ligation carries a clinically and statistically significant higher risk of complications than surgical excision [9].

The purpose of this paper is to present two cases that associate all these alterations, without a syndromic relationship: the first case presents ankyloglossia, syndactyly and polydactyly; the second case presents ankyloglossia and syndactyly. This could lead to a more detailed assessment of newborns who present these alterations and create a better treatment plan, with a multidisciplinary medical team.

## Case presentation

Case 1

**Figure 1:**
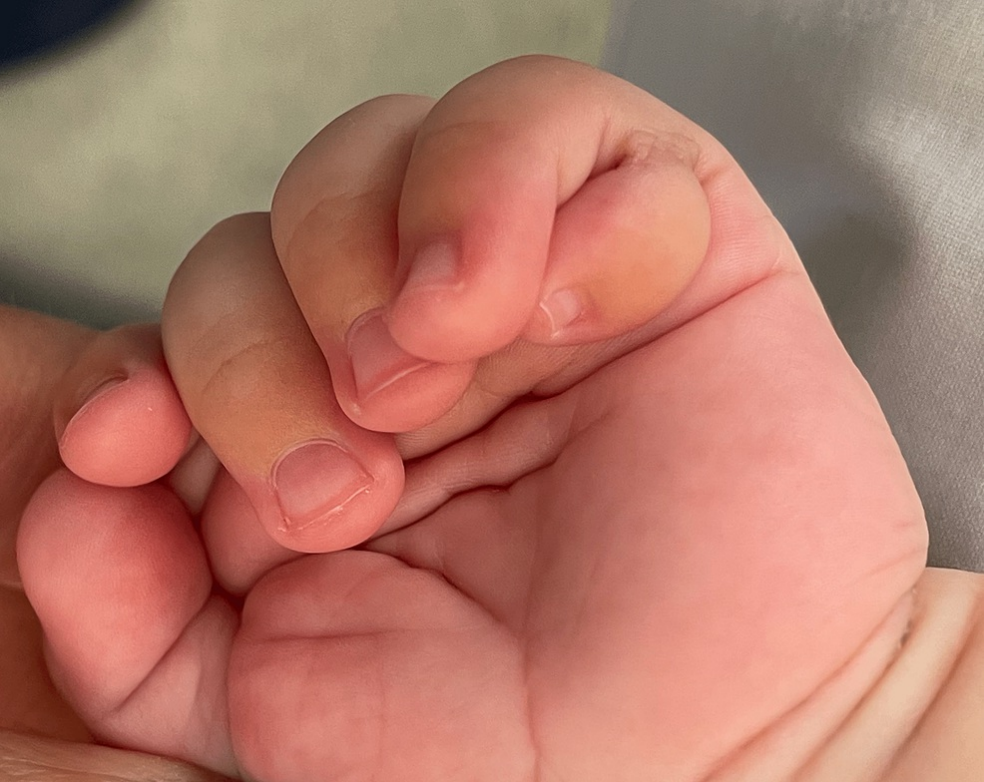
Type B ulnar polydactyly in left hand (Case 1)

**Figure 2:**
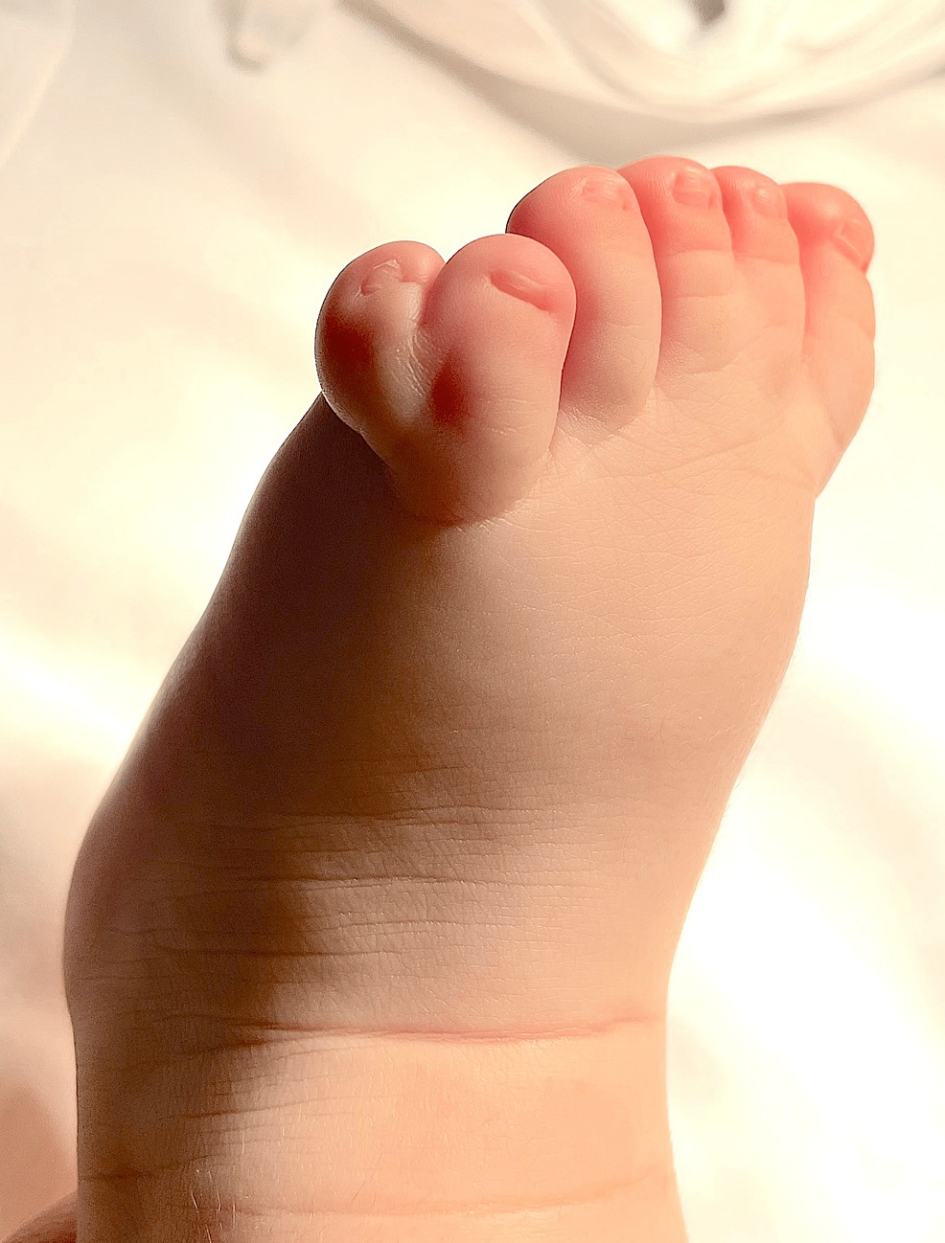
Type A ulnar polydactyly in the left foot (Case 1)

A four-month-old Caucasian boy presented to a private dental hospital in Oporto (Portugal), for an evaluation of the lingual frenulum, referred by his pediatrician. His medical history was positive for syndactyly in his second and third, fifth and sixth left foot fingers. The patient had also a type A ulnar polydactyly in the left foot, and a type B ulnar polydactyly in both hands (Figures 1, 2). His primiparous mother had a regular pregnancy and delivery at 38 weeks, with a family history of syndactyly and polydactyly. It was completed the golden hour with breastfeeding skin-on-skin; however, this was conducted with several difficulties until the weaning at three months old.

 

On the appointment day, the baby was already on bottle-exclusive formula milk, and, during the lactation session, some alterations were observed in suckling and swallowing, snapback, loss of vacuum, loss of milk by the labial commissure, gagging during deglutition, exaggerated pharyngeal reflex (GAG) and gastroesophageal reflux.

**Figure 3:**
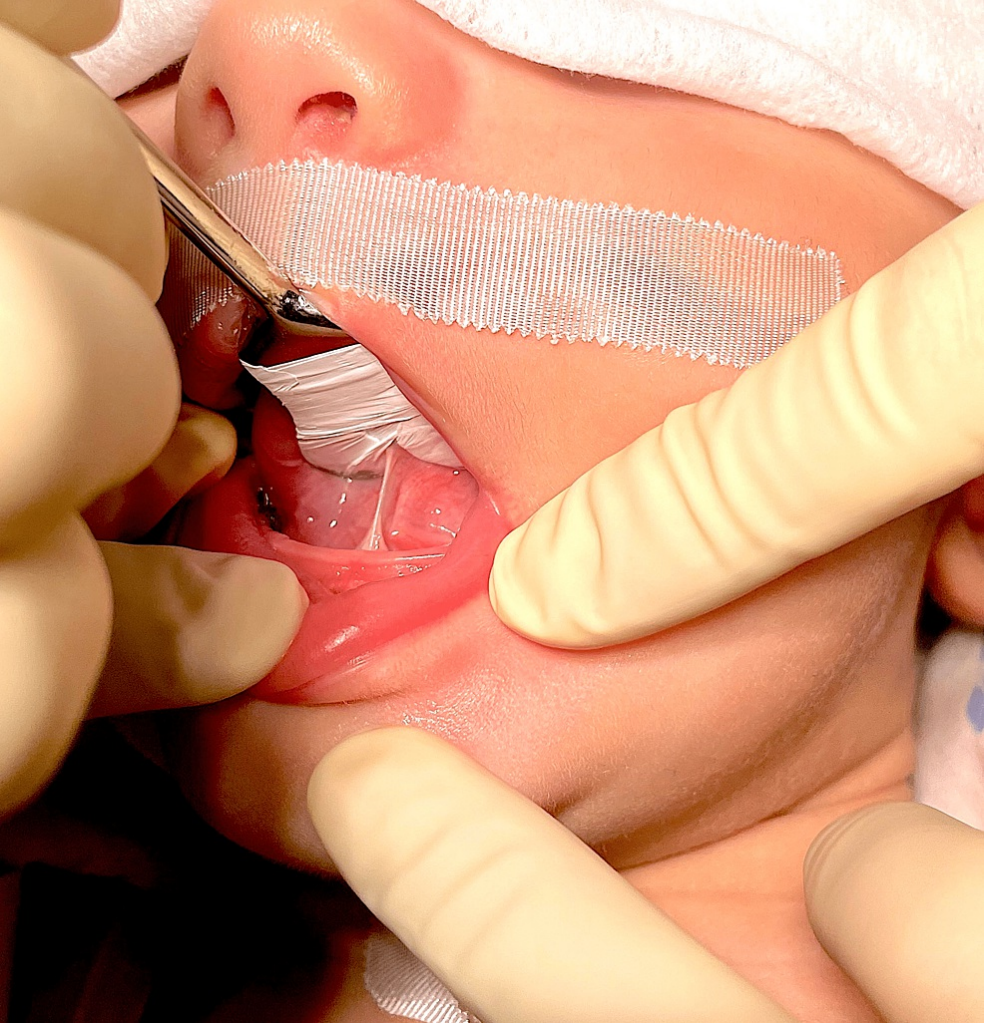
Ankyloglossia (Case 1)

The oral cavity was observed and two protocols for evaluation of the lingual frenulum were applied: (1) Hazelbacker Protocol (Assessment Tool for Lingual Frenulum Function™) (ATLFF) [10] and (2) Coryllos Classification [11]. The ankyloglossia was classified as ATLFF 8 in function and 4 in appearance, and as Coryllos grade 1, with indication for lingual frenotomy (Figure 3).

After discussing treatment options with the family and obtaining informed consent, it was decided to perform polydactyly removal and lingual frenotomy at the same anesthetic moment, with the objective of simplifying and offering less stress to the baby. Regarding the syndactyly correction, it was postponed until after three years of age in accordance with the current guidelines for pediatric anesthesia for all non-emergent routine procedures [12].

**Figure 4:**
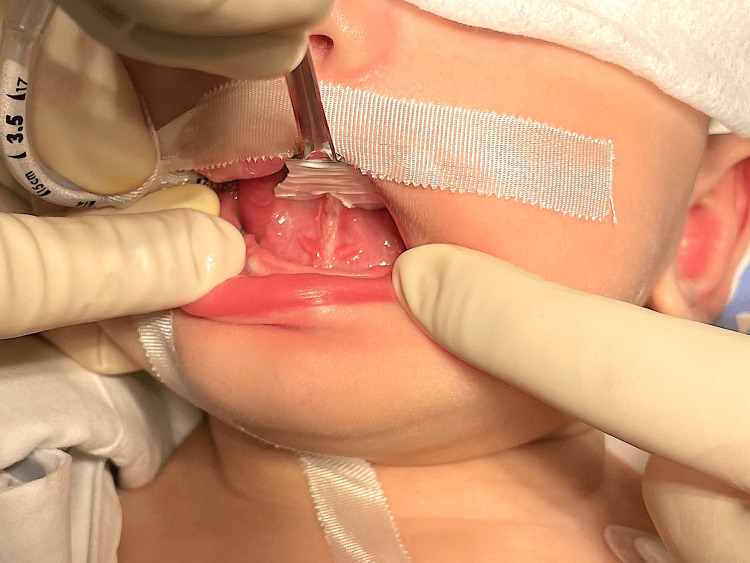
Tongue after diode laser frenotomy: diamond-shaped surgical wound (Case 1)

**Figure 5:**
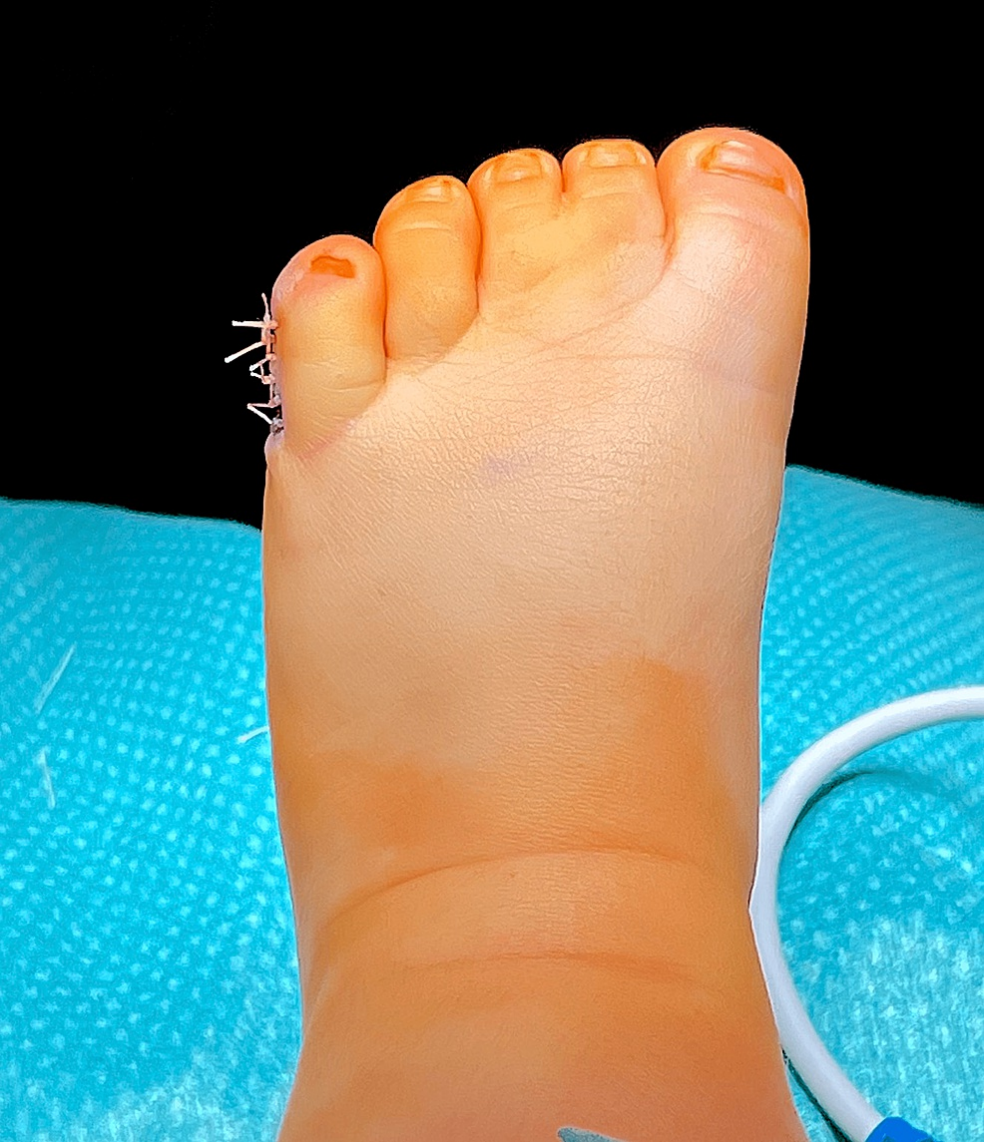
Left foot after polydactyly correction (Case 1)

**Figure 6:**
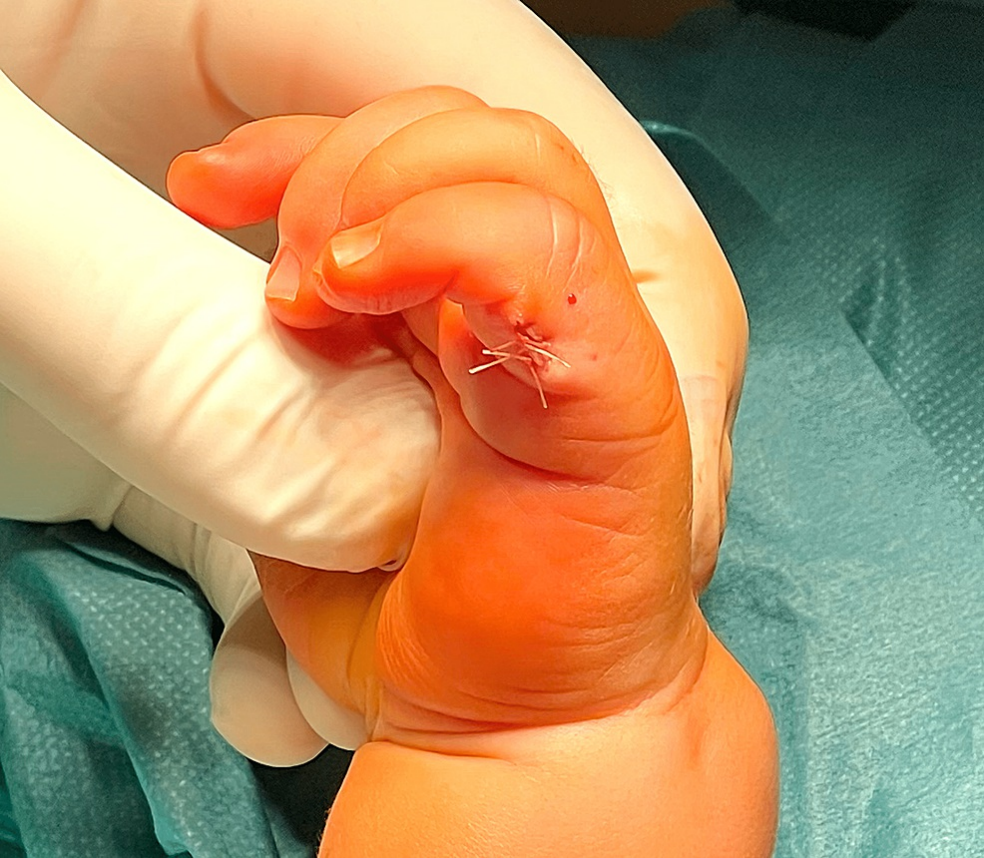
Left hand after polydactyly correction (Case 1)

In both corrections, surgery was completed in one hour under general anesthesia and proceed without complications. The lingual frenotomy was performed by a diode laser in a simple frenotomy technique [13]: the lingual frenulum was dissected from the mucous layer to the muscular fascia. Care was taken to avoid damage to the lingual muscles, the blood vessels, the lingual nerve and the Wharton's duct. Tongue mobility was checked and a diamond-shaped surgical wound was obtained. Due to the excellent cauterization, achieved by the diode laser, no sutures were necessary (Figure 4). For the finger removal, a #12 scalpel and an electric scalpel were used (Figures 5, 6), the surgical wounds were sutured with Vicryl Rapid®, composed of 90% glycolide and 10% L-lactide copolymer. It was recommended analgesic medication for the first three days (oral paracetamol) and there were no postoperative complications. Follow-up was scheduled one week after surgery.

 

Case 2

**Figure 7:**
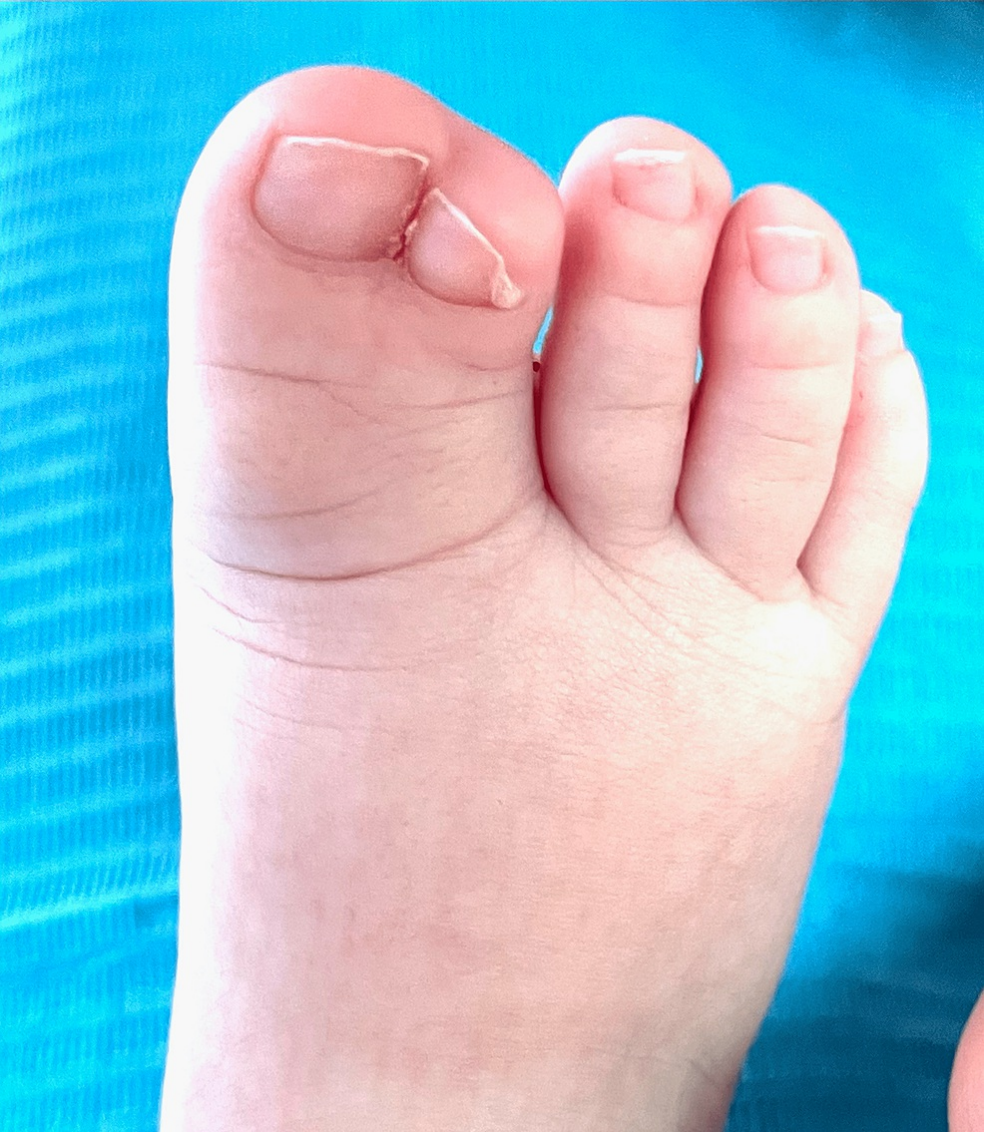
Right foot with syndactyly the first and second fingers (Case 2)

**Figure 8:**
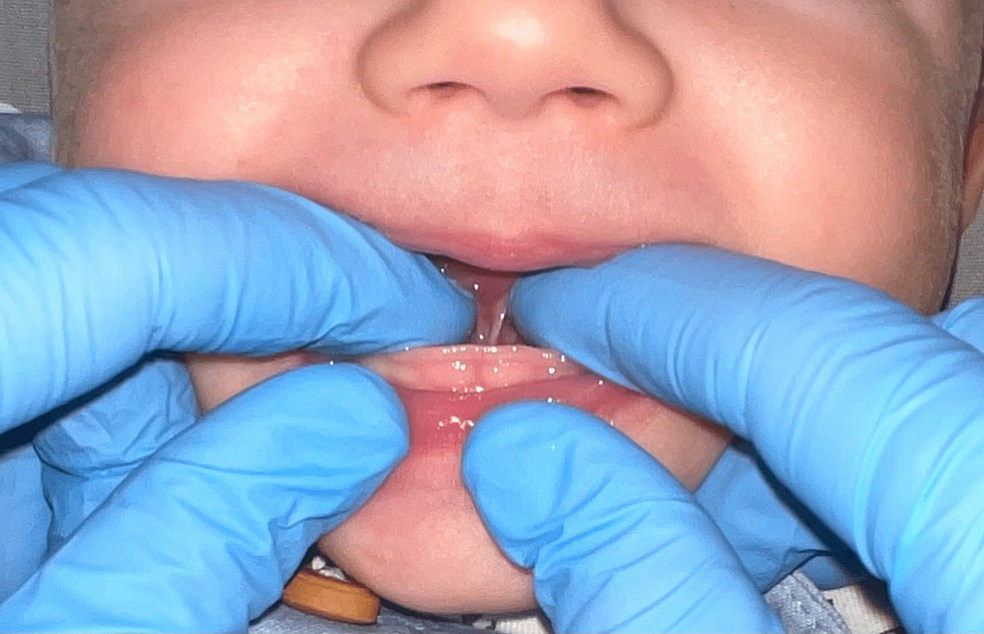
Ankyloglossia (Case 2)

A three-month-old Caucasian boy presented to a private dental hospital in Oporto (Portugal), for an evaluation of the lingual frenulum, referred by his family doctor. His medical history was positive for syndactyly in his first and second left foot fingers (Figure 7). His primiparous mother had a regular pregnancy and an eutocic delivery, without complications. The oral cavity was observed and the same protocols for the evaluation of the lingual frenulum were applied: ATLFF [10] and Coryllos [11]. The ankyloglossia was classified as ATLFF 12 in function and 8 in appearance, and as Coryllos grade 3 (Figure 8), with indication for lingual frenotomy.

Breastfeeding ran into some difficulties from the beginning. The mother felt pain when breastfeeding and the baby had episodes of gagging and clicking during suckling and swallowing. In the appointment, the baby's latch and breastfeeding posture was corrected by an International Board Certified Lactation Consultant (IBCLC), which led to an efficient breastfeeding with no pain felt by the mother. The baby was then referred to speech therapy and to the pediatric orthopedics appointment.

## Discussion

In the literature [14], some associations were found relating polydactyly, syndactyly, and ankyloglossia, such as oral-facial-digital syndromes (OFDS). OFDS are a rare group of developmental disorders that affect the face, oral cavity, and fingers. There are 14 types of OFDS and the first, type 1, was reported by Papillon-Léage and Psaume, in 1954, which is the most common type observed [14]. However, other rarer syndromes associate similar alterations, such as the Laurence-Moon-Biedl syndrome or Pallister-Hall syndrome [8]. Concerning in association between ankyloglossia and syndactyly, without a syndromic relationship, no studies were found in our research.

Despite the increasing number of articles related to ankyloglossia or “tongue-tie,” it remains uncertain how effective the classification assessment tools are in diagnosing symptomatic tongue-tie and fulfilling lingual frenectomy criteria [2]. For example, in Norway, there was an increase of seven times more diagnoses of ankyloglossia, and 13 times more lingual frenotomy surgeries, between 2008 and 2019 [15]. These numbers reinforce the importance of further research, as, to date, there are no standard criteria for diagnosis and treatment [15].

On the other hand, a systematic review [2] was recently conducted to identify a possible significant correlation between pre-treatment statistical scores of tongue-tie and post-treatment outcomes in breastfeeding and speech, by a group of researchers from the University of Adelaide, Australia. It was found that patients may improve their function related to breastfeeding and speech. However, due to the lack of consensus regarding pre-treatment assessment methods, diverse outcome measures, and poor study design and methodology, precise quantification of any improvement cannot be calculated. In this article, the most used classification systems were the ATLFF [10] and Coryllos [11], nevertheless, the ATLFF protocol is the only one that can suggest surgical intervention [2]. The AAPD reinforces that surgical treatment of ankyloglossia needs to be individualized and discussed with the family and within a multidisciplinary team of health professionals [1].

In the first case report, in addition to the two protocols applied (ATLFF and Coryllos), both anatomy and lingual function were evaluated, as well as signs and symptoms associated with ankyloglossia. After the lingual frenotomy, the patient performed speech therapy exercises to ensure optimal healing and lingual mobility. A few weeks after surgery, in the control appointment, with 5 months old, several improvements were observed in terms of oral functionality and motor skills. The patient began to improve free movement (rolling), lifting and holding of the head, no longer losing vacuum in the bottle teat, nor losing milk through the labial commissure. There were no more episodes of choking, the GAG normalized, and the gastroesophageal reflux improved. The rehabilitation work of the physiotherapy, osteopathy, and speech therapy team may have contributed to the outcome.

Regarding the second case, despite the ATLFF protocol indicating surgical intervention, it was postponed, as the difficulties with breastfeeding were overcome and a single anesthetic moment is preferred. However, a reassessment is scheduled before the introduction of complementary food, as well as a follow-up by an experienced speech therapist. If syndactyly correction is to be performed at a later time and if tongue functional alterations are, meanwhile, detected, lingual frenotomy will be anticipated and performed under local anesthesia.

## Conclusions

In conclusion, in the presence of polydactyly and/or syndactyly, ankyloglossia may also be present, without being associated with any type of syndromic disease. This finding may be useful, as polydactyly and syndactyly are more easily diagnosed, and their presence may lead health professionals to search for the presence of ankyloglossia, which is usually associated with some signs and symptoms, mainly related to breastfeeding, at earlier ages, or speech difficulties. Early diagnosis and un­derstanding of ankyloglossia are important, as it allows to prevent early weaning in breastfeeding infants and other possible complications in the future.
